# What do the patients with medication overuse headache expect from treatment and what are the preferred sources of information?

**DOI:** 10.1007/s10194-011-0298-4

**Published:** 2011-02-20

**Authors:** S. B. Munksgaard, M. Allena, C. Tassorelli, P. Rossi, Z. Katsarava, L. Bendtsen, G. Nappi, R. Jensen, The Comoestas Consortium

**Affiliations:** 1 Danish Headache Centre, Department of Neurology, Glostrup Hospital, Faculty of Health Sciences, University of Copenhagen, Glostrup, Denmark; 2 University Centre for the Study of Adaptive Disorders and Headache (UCADH), University of Pavia, Pavia, Italy; 3 Headache Science Centre, IRCCS “National Neurological Institute C. Mondino” Foundation, Pavia, Italy; 4 Department of Neurology, University of Duisburg-Essen, Essen, Germany

**Keywords:** Medication overuse headache, Expectations, Sources of information, Education

## Abstract

Lack of knowledge on patients’ expectations to treatment may lead to misunderstandings and prevent successful outcome. Presently, treatment of medication overuse headache (MOH) leads to improvement in up to 75% of patients, but the relapse rate may exceed 40%. This study aimed to evaluate the preferences on information and expectations to treatment in patients entering a treatment programme for MOH. A questionnaire on patients’ needs and preferences on information and expectations was distributed to 65 MOH patients from specialized headache clinics in Italy, Germany and Denmark. A total of 75% selected personal verbal information as their primary need, significantly higher than the percentage of patients who selected leaflets and website information 35 and 35%, respectively (*p* < 0.001). Telephone and E-mail consultation was requested by 59 and 48%, respectively. The information source preferred was again personal verbal information (82%), significantly higher than all other information sources (*p* < 0.001). In decreasing order, patients preferred telephone consultation (48%), E-mail consultation (44%), website information (41%), and leaflets (33%). 51% expected their headache to be cured, 71 and 57% requested effective prevention and fast relief of the headache episodes. 80 and 75%, respectively expected reduction in frequency and intensity. A total of 64% expected information about self-management and 52% expected to receive education on their headaches. The study demonstrates that patients in specialized headache centres prefer personal information, that expectations are very high, and that education and information are important. Providing the right information and thus give patients realistic expectations might enhance compliance and improve outcome.

## Introduction

Chronic headache is frequently associated with a high degree of psychosocial stress and patients with chronic headache are more often dissatisfied with medical and therapeutic management than persons with episodic migraine [1]. Lacking knowledge on what patients expect from the treatment programme and outcome may lead to misunderstandings and prevent a successful outcome [2, 3].

Many studies focus on objective and easily measured outcomes such as medication use and headache days rather than focusing on the patients’ preferences and subjective opinion of the treatment. For example the efficacy of most migraine drugs is evaluated with pain relief at 2 h as the primary end point even though all patients defined rapid pain relief as pain relief happening within 1 h [4]. This may lead to dissatisfaction with treatment.

Medication overuse headache (MOH) is a chronic secondary headache. The patients suffer from headache more than 15 days/month and take medication to relieve the headache 15 days/month or more for simple analgesics or 10 days/month or more for opioids, triptans, ergots, or combination analgesics [5].

The present treatment strategy of MOH is employed with emphasis on abrupt cessation of the overused drug and initiation of a prophylactic treatment for the primary headache which in the first place led to the medication overuse. This is a rather long and complicated course, but nevertheless it has been shown in previous studies that up to 75% of patients improve after detoxification [6]. For instance, in the study of Zeeberg et al. approximately 70% out of 216 MOH patients demonstrated a 50% reduction in headache frequency and intensity after treatment. However, it is noteworthy that up to 40% of successfully detoxified MOH patients may relapse into medication overuse within a year [6, 7]. Many determinants may be involved in the relapse and a recent paper from the Pavia Group has shown that an adapted follow-up (CARE approach), simply based on fixed control visits with the same doctor, yields a lower rate of relapse at 1 year (22%) [8]. This positive result may partly be caused by the fact that before the withdrawal the patients knew exactly when the follow-up visits would take place and they knew the doctor who was responsible, i.e., they knew what to expect and their expectations were met.

We therefore hypothesize that if patients’ expectations and preferences for treatment are realistic and these are met, patient compliance and satisfaction with treatment might increase.

On this background, the present survey was performed as a pilot study in three European Countries within the COMOESTAS project^1^ for evaluating MOH patients preferences and expectations regarding their disease management in terms of (i) preferred sources of information and (ii) treatment programme.

## Methods

A draft questionnaire was created to measure the needs and expectations of patients suffering from MOH. Translation of the questionnaire into local languages (Italian, Danish and German) was performed according to the forward–backward–forward methodology with the participation of an English and a native language speaker. Thereafter, the questionnaire was tested on 10 consecutive MOH patients in the tertiary headache centre in Pavia, in order to evaluate understanding of questions and scoring system by the patients.

A final version containing questions on patients’ preferences regarding information and expectations for the treatment was then obtained and translated into German, Italian and Danish. This was distributed to 65 consecutive MOH patients attending the tertiary headache centres of Essen, Pavia and Glostrup.

The patients were all asked to fill in the questionnaire on the day of their first consultation leading to the diagnosis of MOH, before being seen by the doctor.

**Fig. 1:**
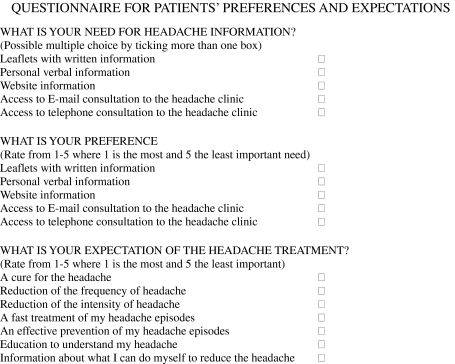
Questionnaire

The questionnaire included three sections: (1) the patients’ needs for information, (2) the patients’ preferences regarding information and (3) the patients’ expectations regarding the treatment of MOH (Fig. 1).

In Sect. 1, the patients had to tick all the sources of information they needed from a list of five items. In Sect. 2, they had to rate their preference for each of these five items with a number from 1 to 5, where the items rated 1 were the most and 5 the least important. In Sect. 3, the patients had to rate each of the seven items on expectations with the numbers 1–5, according to the same scoring system described for Sect. 2. Hence, it was possible to rate more than one expectation or source of information as the most or least important.

## Statistics

Items rated 1 or 2 in Sect. 2 were counted as a preferred sources of information and in Sect. 3 as the most important expectations as regards treatment. Unrated items were excluded from the calculations. To compare frequencies between the countries, between needed or preferred sources of information, and between different expectations we used the χ^2^ test for samples with more than five in each group and Fisher’s exact test for smaller samples. The level of significance was set to a *p* value of 0.05.

## Results

All 65 patients filled in the questionnaire as requested. Fifteen of the questionnaires had one or more missing values in Sect. 2 and 3, 14 from Denmark, 1 from Italy.

The patients’ needs for information sources are shown in Table 1. Personal verbal information was selected by 75%. This percentage was significantly higher than the percentage of patients who selected leaflets and website information (35 and 35%, respectively, *p* < 0.001). The second most frequently requested source of information was access to telephone consultation (59%), while access to E-mail consultation only was indicated by 48%. When comparing countries, there was a statistically significant difference between the 15% of patients from Italy and 52% of patients from Denmark who required leaflets (*p* = 0.013), but otherwise there were no differences between types of sources of information preferred by the patients.

**Table 1:** What is your need for headache information?

	Italy	Germany	Denmark	Total
No. of patients	20	20	25	65
Leaflets with written information	**15.0%** (0–30.7)	35.0% (14.1–55.9)	**52.0%** (32.4–71.6)	35.4% (23.8–47.0)
Personal verbal information	75.0% (56.0–94.0)	70.0% (49.9–90.1)	80.0% (64.3–95.7)	75.4% (64.9–85.9)
Website information	35.0% (14.1–55.9)	35.0% (14.1–55.9)	36.0% (17.2–54.8)	35.4% (23.8–47.0)
Access to E-mail consultation to the headache clinic	50.0% (28.1–71.9)	35.0% (14.1–55.9)	56.0% (36.54–75.5)	47.7% (35.6–59.8)
Access to telephone consultation to the headache clinic	50.0% (28.1–71.9)	65.0% (44.1–85.9)	60.0% (40.8–79.2)	58.5% (46.5–70.5)

The 95% confidence interval is in brackets

The preferences for information sources (Table 2) were distributed very similarly to the needs. The source of information preferred by most patients was personal verbal information, which was selected by 82%. This was a statistically significantly larger percentage than all other sources of information (*p* < 0.001). In decreasing order, patients preferred access to telephone consultation (preferred by 48%), access to E-mail consultation (44%), website information (41%), and leaflets (33%).

**Table 2:** What is your preference of headache information?

	Italy	Germany	Denmark	Total
No. of patients	20	20	25	65
Leaflets with written information	25.0% (6.0–44.0)	30.0% (9.9–50.1)	42.9% (21.7–64.0)	32.8% (21.4–44.2)
Personal verbal information	73.7% (53.9–93.5)	90.0% (76.9–100)	82.6% (67.8–97.5)	82.3% (72.7–91.8)
Website information	36.8% (15.2–58.5)	55.0% (33.2–76.8)	30.0% (12.0–48.0)	40.7% (28,1–53,2)
Access to E-mail consultation to the headache clinic	42.1% (19.9–64.3)	40.0% (18.5–61.5)	50.0% (30.4–69.6)	43.9% (31.0–56.7)
Access to telephone consultation to the headache clinic	45.0% (23.2–66.8)	60.0% (38.5–81.5)	40.9% (21.6–60.2)	48.4% (35.9–60.8)

The 95% confidence interval is in brackets

The patient’s expectations from treatment are illustrated in Table 3. Most patients were expecting a reduction in the severity of the disease (reduction in frequency by 85% and in intensity by another 82%), while a significantly lower percentage of patients was expecting a cure for headache (59%) or education to better understand their disease (58%). 77% of subjects expected an effective prevention of their headache episodes, while 64% expected an acute treatment working fast. It is noteworthy that almost three out of four patients expected to receive information about strategies to actively combat headache. This need was particular felt by 90% of the German patients and by 65% of the Italian and by 63% the Danish patients.

**Table 3:** What are your expectations of the headache treatment?

	Italy	Germany	Denmark	Total
No. of patients	20	20	25	65
A cure for the headache	80.0%* (62.5–97.5)	45.0%* (23.2–66.8)	50.0% (30.4–69.6)	58.9%^†‡^ (46.0–71.8)
Reduction in the frequency of headache	85.0% (69.4–100)	90.0% (76.9–100)	81.0% (65.6–96.3)	85.2% (76.3–94.1)
Reduction in the intensity of the headache	85.0% (69.4–100)	85.0% (69.4–100)	75.0% (58.0–92.0)	81.6% (71.9–91.5)
A fast treatment of my headache episodes	70.0% (50.0–90.1)	60.0% (38.5–81.5)	61.1% (42.0–80.2)	63.8%^†^ (51.4–76.2)
An effective prevention of my episodes	75.0% (56.0–94.0)	85.0% (69.4–100)	72.7% (55.2–90.2)	77.4% (67.0–87.8)
Education to understand my headache	50.0% (28.1–71.9)	75.0% (56.0–94.0)	47.4% (27.8–66.9)	57.6%^†‡^ (45.0–70.2)
Information about what I can do myself to reduce the headache	65.0% (44.1–85.9)	90.0% (76.9–100)	63.2% (44.8–82.1)	72.9% (61.5–84.2)

The 95% confidence interval is in brackets

* *p* < 0.05 between countries, ^†^ *p* < 0.05 compared with reduction in the frequency of headaches and reduction in the intensity of headaches, ^‡^ *p* < 0.05 compared with an effective prevention of my episodes

As regards the country comparison, Italian patients seemed more optimistic as they expected a cure from headache in 80% of cases when compared with the German (45%) (*p* = 0.048) or Danish (50%) (*p* = 0.082) patients. There were no other statistically significant differences between the countries as regards the expectations.

## Discussion

From this pilot evaluation we can derive that for MOH patients the preferred channel of information is represented by personal contact, which was indicated by the majority of patients, both as face-to-face personal verbal information and telephone consultation. This pattern was expected as our patients are from tertiary headache centres but the pattern was similar in the three countries. As regards some of the “alternative” modalities of information (leaflets and website), these were selected only by 1/3 of patients, with a very low percentage for leaflets in Italy. MOH is a severe form of headache and most of the patients consulting third-level headache centres, like the three involved in this study, have a long history of headache and of unsuccessful treatments. The personal contact will probably make the patients feel more confident as they can instantly ask questions and get explanations on the disease or prescriptions.

We were surprised to find than nearly 2 out of 3 patients did not like indirect or innovative sources of information (leaflets and websites, respectively), even though these sources are always available and ready to use at any time. In the case of leaflets, this is likely due to fact that they usually feature general, non specific content and are therefore of limited help for the patients. In the case of websites, the internet gives access to a huge quantity of information, which may be either too general or non controlled or, worse, misleading due to the substantial “absence of a governing body or authority that serves a gate-keeping function for web publications” mentioned by Borowitz and Wyatt [9] more than 10 years ago. If a website was created by professionals and the patients were told this, then this might be preferred by a higher number.

It is interesting that nearly 50% of patients selected E-mail consultations as preferred modality of information, which represents a clear opening to a possible alternative modality of consultation in many countries. General accept and IT-implementation are, however, essential in all European countries as it has been estimated that in Europe the internet penetration, and consequently, the percentage of patients who have access to E-mail technology is only slightly above 50%.^2^

E-mail communication is convenient both for the patients and the health professionals. We have been testing it within the COMOESTAS study and the preliminary evaluation suggests that it satisfies the patients, who can detail their problems and receive feed-back within a few hours of the request without the need to spend time going to the centre or staying on the phone for contacting the nurse or the doctor. The physician, on the other hand, can reply in the most convenient time, when he/she has had access to health records of the patient.

In the treatment of MOH it is crucial that the patients avoid overuse of headache medication again in the early phases of detoxification, despite the often daily and severe headaches [10]. One limitation of this study is that, due to the small number of subjects, we could not perform a stratification analysis of data. Another limitation is the results cannot be translated directly to all MOH patients as our patients are included from tertiary centres, and thereby represent selected patients that may have failed prior treatment programmes within first or second line. On the other hand, it is very important to optimize their treatment programmes, as these patients represent the severely affected patient population with a high burden of disease and maybe also an increased risk of relapse. Thirdly, the relative high number of missed replies in Denmark but not in Italy and Germany indicates a methodological variation between countries, although we were unable to identify whether it was a cultural variation or lack of proper instruction to complete all questions. Thus, our questionnaire may have benefited from additional validation tests but we have deliberately eliminated validation against direct interview to avoid patient-to-doctor-bias.

However, it is likely that alternative modalities of communication may be preferred by and will be more effective in selected subgroups of subjects, representing a new and interesting field of headache research and care. Likewise, international E-mail communication may also be implemented to countries with limited access to specialized headache care or for rare headache disorders. Indeed a previous study has shown that the use of internet and SMS is welcome in healthcare delivery amongst young people and people with a higher education [11]. The percentage of people preferring web-based sources of information as E-mail and websites will probably increase with time.

As for treatment, patients showed high expectations. More than half expected to be cured from their headache even though almost no patients will experience a total absence of headache after detoxification [6]. This stresses the need for detailed and realistic information before onset of the treatment programme emphasizing that headache can be improved but not yet cured. This is important as Garrity et al. [3] reported that patient compliance with treatment decreases when expectations are not met. Giving proper and detailed information on the treatment programme and what outcome can be expected may also avoid misunderstandings and may also improve the success rate, as realistic expectations to the treatment may improve the patients’ satisfaction and enhance the chance of a successful outcome [2].

The high percentage of patients expecting to obtain a reduction in the severity of their disease (more than 80%)—and the fact that Sect. 3 was conceived as a multiple choice—seems however to suggest that preference for “a cure for headache” item actually represented a wish, while the real(istic) expectation was actually represented by an improvement in headache intensity and frequency.

Interestingly, more than half of the patient population considered it important to receive information and education about self-management and understanding of headache. Educating the patients may qualify them in becoming active partners in their own treatment and this might increase compliance with the treatment as the patients experience a responsibility for their own treatment.

The study was designed as a pilot study in three specialized headache centres and the applied methodology and results do not allow definite conclusions. Nonetheless, we believe that this study is important for several reasons:

It demonstrates that personal information by telephone or face to face is preferred by most patients, that patients expectations are very high, which points to the need for information about realistic outcome, and that education and information is important to most patients. Furthermore, this study helps to clarify what is important to the patients in specialized clinics, and underline that such endpoints should be added to the strictly objective outcomes such as headache days, medication use and absence rates.

So in conclusion, MOH is a challenge for physicians and new strategies need to be devised to improve long-term outcome of this disease. For doing this, it is important to take into consideration patients’ preferences and expectations and to give detailed and realistic information about what can and what cannot be achieved from a treatment program. Giving the right information and educating the patients might enhance compliance and over time improve treatment outcome.

## Appendix

G. Sances, G. Sandrini, F. Blandini (IRCCS “Neurological Institute C. Mondino” Foundation, University Centre for Headache and Adaptive Disorders, Pavia), M. Lainez, B. Lopez (Fundación de la Comunidad Valenciana para la Investigación Biomédica, la Docencia y la Cooperación Internacional y para el Desarrollo del Hospital Clínico Universitario de Valencia—Spain), Z. Katsarava, M. Rapsch (Department of Neurology, University Hospital of Essen), J. Leston, M.T. Goicoichea (Fundacion para la Lucha contra las Enfermedades Neurologicas de la Infanzia, Buenos Aires—Argentina), R. Fadic, B. Shand (Pontificia Universidad Catolica de Chile, Santiago—Chile), S. Spadafora, M. Osa (Unversidad Isalud, Buenos Aires—Argentina), A. Stoppini, M. Pagani (Consortium of Medical and Informatic Bionegeneering, Pavia—Italy).

## Footnotes

1 COMOESTAS Project—VII FP–EC contract number 215366 (COMOESTAS) FP7—Thematic priority ICT—http://www.comoestas-project.eu. The COMOESTAS project’s aim is to develop an electronic system for monitoring MOH patients to facilitate the communication between patient and doctor, to evaluate the present treatment of MOH, and to enhance cooperation between European and Latin American countries.

2
http://www.internetworldstats.com/stats4.htm
